# Validation of a French Version of the Quality of Life “Celiac Disease Questionnaire”

**DOI:** 10.1371/journal.pone.0096346

**Published:** 2014-05-02

**Authors:** Jacques Pouchot, Carole Despujol, Georgia Malamut, Emmanuel Ecosse, Joël Coste, Christophe Cellier

**Affiliations:** 1 Service de médecine interne, hôpital européen Georges Pompidou, Assistance Publiques – Hôpitaux de Paris, Université Paris Descartes, Sorbonne Paris Cité, Paris, France; 2 APEMAC, EA 4360, Université de Lorraine, Université Paris Descartes, Sorbonne Paris Cité, Paris, France; 3 Service de gastroentérologie, hôpital européen Georges Pompidou, Assistance Publiques – Hôpitaux de Paris, Université Paris Descartes, Sorbonne Paris Cité, Paris, France; University of Texas Health Science Center at Houston, United States of America

## Abstract

**Background and Objective:**

Celiac disease (CD) is a common chronic autoimmune disorder. Both the manifestations of the disease and the burden of the compulsory life-long gluten-free diet (GFD) have been shown to be associated with impairment of health-related quality of life. The objectives of this study were to provide a cross-cultural adaptation of the specific quality of life “Celiac Disease Questionnaire” (CDQ) and to analyze its psychometric properties.

**Materials and Methods:**

A cross-cultural French adaptation of the CDQ (F-CDQ) was obtained according to the revised international guidelines. The questionnaire was administered at baseline to 211 patients with biopsy proven CD followed-up in a single tertiary referral centre. The questionnaire was also administered after 7 days and 6 months. Reliability (intraclass correlation coefficients (ICC), Cronbach's alpha and Bland and Altman graphical analysis), validity (factorial structure and Rasch analysis, convergent validity), and responsiveness (effect size) of the F-CDQ were studied.

**Results:**

The reliability of the F-CDQ was excellent with ICC and Cronbach's alpha coefficients being between 0.79 and 0.94 for the four subscales and the total score. The factorial structure and the Rasch analysis showed that the four dimensions of the original instrument were retained. Correlations with external measures (a generic measure of quality of life, an anxiety and depression instrument, a self-assessed disease severity, and clinical manifestations) were all in the expected direction confirming the validity of the instrument. Responsiveness was studied and effect sizes ≥0.20 were demonstrated for most of the subscales for patients who reported improvement or deterioration after 6 months.

**Conclusion:**

The F-CDQ retains the psychometric properties of the original instrument and should be useful in cross-national surveys and to assess outcome in clinical trials involving patients with CD.

## Introduction

Celiac disease (CD) is a chronic inflammatory disorder of the small bowel caused by gluten ingestion in genetically susceptible individuals. It is an autoimmune disorder that is characterized by small bowel villous atrophy and intra-epithelial infiltration by lymphocytes associated with specific antibodies in serum. Surveys involving screening of the general population based on serological testing have shown that CD is a common disorder, and the prevalence has been estimated to be approximately 1% in Europe and the USA [1].

The typical presentation of CD includes diarrhoea, abdominal pain, and weight loss, but the clinical spectrum of CD is wide and extra-intestinal manifestations, including iron-deficiency anaemia, arthralgia, osteoporosis, and even infertility or miscarriage are encountered in more than 50% of CD patients, and, indeed, may reveal the disease [1]–[3]. Some patients are “asymptomatic” with no apparent symptoms and are diagnosed only by screening of the general population [4].

The only currently available treatment is a life-long and strict gluten-free diet (GFD); this allows control of the clinical manifestations, normalisation of the intestinal mucosa, and the disappearance of disease-specific antibodies from the serum [1]–[3]. Also, there is evidence that a GFD protects patients with either symptomatic or asymptomatic CD against the occurrence of small intestinal lymphoma. The benefits of being free of clinical manifestations of CD are, at least in part, counterbalanced by the burden of the GFD, which is difficult to follow in the long term, especially for the asymptomatic individuals. Indeed, a GFD limits pleasure and socialization associated with food, and also has financial consequences. These issues may be particularly significant for asymptomatic patients identified by screening. Also, a GFD may have different effects in different countries, as diet styles may differ (i.e. the popularity of the French baguette in France).

Patient-reported outcomes that capture self-perceived health concerns have become key measures over recent decades, and health-related quality of life (HRQoL) instruments are commonly used in epidemiology, clinical trials, and for routine follow-up of patients. However, only limited data are available describing the quality of life of patients with CD. Most previous studies used the generic medical outcome study short-form 36 items (MOS-SF36) [5] and reported that CD is detrimental for HRQoL and that this effect is reduced by a GFD [6]–[9]. In the study by O'Leary et al. [6] CD patients with gastrointestinal symptoms had lower (worse) MOS-S36 scores both than those without and than patients who were following a GFD. In another study, unsatisfactory compliance with the GFD was found to be associated with a secondary deterioration of HRQoL [7]. In the study by Johnston et al. [8] patients with typical CD had significantly lower scores than controls for four out of the eight subscales of the MOS-SF36 (“General Health” (GH), “Vitality” (VT), “Role emotional”, and “Mental Health”); the scores for two of these scales (GH and VT) improved significantly after one year on a GFD [8]. However, in contrast to these results, another study showed that despite strict adherence to GFD, ten years after diagnosis most patients failed to achieve a quality of life similar to that of the general population [9].

In a national survey of patients with CD in the USA in 2011, using a single transition item, 77% of the patients reported an improvement in their quality of life following the diagnosis of CD [10]. A Canadian population-based survey in 2003 showed that summary scores for physical and mental function derived from the MOS-SF12 for most CD patients on a GFD were similar to those of the general Canadian population, and had improved since starting the GFD [11], [12]. A German national survey also demonstrated the detrimental consequences of CD on HRQoL as measured by MOS-SF36, and the improvement associated with compliance with a GFD [13].

Although useful because it is widely used, the MOS-SF36 instrument was designed to measure HRQoL in the context of diverse chronic disorders; it is thus a generic questionnaire, and does not address health problems specifically associated with CD [5]. Recently, a specific HRQoL questionnaire, the “Celiac Disease Questionnaire” (CDQ), was developed for adults patients in German by Häuser et al. [14]. The CDQ explores gastrointestinal symptoms, psychological wellbeing, and social functioning. Items were generated using a literature review and the questioning of patients with CD and gastroenterologists. Psychometric properties that were studied during its development included face validity, factorial structure, internal consistency and test-retest reliability, convergent validity, and discriminant validity.

The objective of our study was to develop a French cross-cultural adaptation of the CDQ and to assess its psychometric properties, including the responsiveness that was not documented in the original version of the questionnaire [14].

## Results

### Characteristics of the patient population

Of the 323 eligible patients with biopsy-proven CD managed in our referral centre, 211 (65.3%) agreed to participate in the study, and provided baseline data. Of these, 144 provided the retest questionnaire at 7 days, and 155, some of whom did not respond to the 7-day retest sent the follow-up assessment at 6 months. Of the 211 patients, 122 provided completed questionnaires at all three time points (57.8%).

The main demographic and clinical characteristics of the patients included in the study are presented in Table 1. The mean age of the patients was 45.1 years and their mean disease duration was 13.3 years. Most were female (78.7%) and highly educated; 57.3% of the patients were married or lived as a couple, and 74.4% reported that they were employed or students at the time of the survey.

**Table 1:** Socio-demographic and clinical characteristics of the patients with celiac disease included in the validation study of the French version of the “Celiac Disease Questionnaire”.

	All patients (N = 211)
Sex	
Female	166 (78.7)
Male	45 (21.3)
Age, years, mean ± SD (range)	45.1±16.6 (18.0–88.0)
Education, N (%)	
Primary	14 (6.6)
Secondary	62 (29.4)
University	133 (63.0)
Employment status, N (%)	
Employed	137 (64.9)
Retired	32 (15.2)
Student	20 (9.5)
Unemployed	19 (9.0)
Marital status, N (%)	
Married or living as a couple	121 (57.3)
Single	65 (30.8)
Widowed/divorced	24 (11.4)
Disease duration, years, mean ± SD (range)	13.3±11.2 (1–53)
Age at diagnosis of celiac disease, years, mean ± SD (range)	32.2±20.3 (0–77)
Manifestations of celiac disease, N (%)	141 (66.8)
Fatigue	95 (45)
Abdominal pain	67 (31.8)
Diarrhoea	44 (20.9)
Bloating	96 (45.5)
Weight loss	15 (7.1)
Associated manifestations, N (%)	
Arthralgia	52 (24.6)
Myalgia	45 (21.3)
Dermatitis herpetiformis or skin rash related to CD	22 (10.4)
Anaemia	21 (10.0)
Aphtosis	14 (6.6)
Self-assessment of disease severity*, median (IQR)	4 (5)
Self-reported strict adherence to a gluten-free diet, N (%)	162 (76.8)
MOS-SF36 (SD)	
Physical functioning	82.6 (23.1)–0.3 (1.3)
Role-Physical	66.6 (39.3)–0.6 (1.4)
Bodily pain	65.1 (26.6)–0.4 (1.2)
General health	57 (23.3)–0.8 (1.2)
Vitality	45.5 (22.4)–0.8 (1.3)
Role-Emotional	59.9 (40.8)–0.8 (1.3)
Social functioning	66.5 (26.4)–0.7 (1.3)
Mental health	56.8 (22)–0.6 (1.2)

*: disease severity was assessed on a visual analogue scale (0–10) with 10 indicating the most severe disease; CD: celiac disease; IQR: interquartile range. The MOS-SF36 scores are presented both as raw score and as age- and sex-adjusted standardised scores using the French general population reference values for age (10-year interval groups) and gender, and expressed as standard deviations (SD).

Despite a high level of self-reported adherence to GFD (76.8%) many patients (141; 66.8%) reported that they were symptomatic at the baseline assessment; the symptoms reported were mainly gastrointestinal, with 20.9% of the patients reporting diarrhoea. Patients were asked to rate their disease severity and the median score was 4 (on a 10 point numerical rating scale with higher score indicating more severe disease).

### Descriptive statistics for the F-CDQ

The F-CDQ was filled-in at baseline by 211 patients. Generally, the questionnaire was completed well and the percentage of missing items was very low: 175 of the 211 (82.9%) questionnaires had no more than two missing items. One item in the “Anxiety” domain related to the risk of inheritance to children had 14 (7%) missing values; for all other items had none to a maximum of 6 (3%) missing values. For items related to sexuality or employment, 26% and 31%, respectively, of the patients answered that the item was not relevant to them.

The mean scores for the four subscales of the instrument and the total HRQoL score, and the percentage of ceiling and floor effects for each subscale and the total score are reported in Table 2. The four subscale scores and total scores were higher (better quality of life) for men than women, but this was statistically significant for the “Emotions” subscale only.

**Table 2:** Baseline subscale and total scores of the French version of the “Celiac Disease Questionnaire” and reliability.

F-CDQ subscale	N	Mean ± SD	Range	Median	IQR	Floor effect (%)	Ceiling effect (%)	ICC (95% CI) *	Cronbach's α (95% CI) **
Emotions	211	53.7±21.9	2–100	52	19	0	0.5	0.90 (0.86–0.93)	0.92 (0.90–0.94)
Social	210	75.6±23.6	0–100	83	12	0.5	15.7	0.94 (0.91–0.96)	0.87 (0.80–0.91)
Worries	210	65.3±22.9	0–100	69	14	0.5	4.8	0.88 (0.83–0.92)	0.79 (0.74–0.84)
Gastrointestinal	211	69.0±19.2	10–100	71	12	0	4.3	0.89 (0.84–0.92)	0.81 (0.77–0.85)
Total score	209	65.9±18.4	10–99	68	13	0	0	0.94 (0.91–0.96)	0.94 (0.92–0.96)

F-CDQ: French version of the “Celiac Disease Questionnaire”; the scores were normalised, ranging from 0 (worst score) to 100 (best score) for each subscale and the total F-CDQ score;

*: ICC computation was limited to the 112 of the 144 patients who participated in the test-retest study and who stated that their health status had not changed significantly between the two assessments;

**: Cronbach's α coefficients were computed from baseline data for the 211 individuals; ICC: intraclass correlation coefficient; 95% CI: 95% confidence interval.

### Psychometric properties

#### Reliability

**Figure 1:**
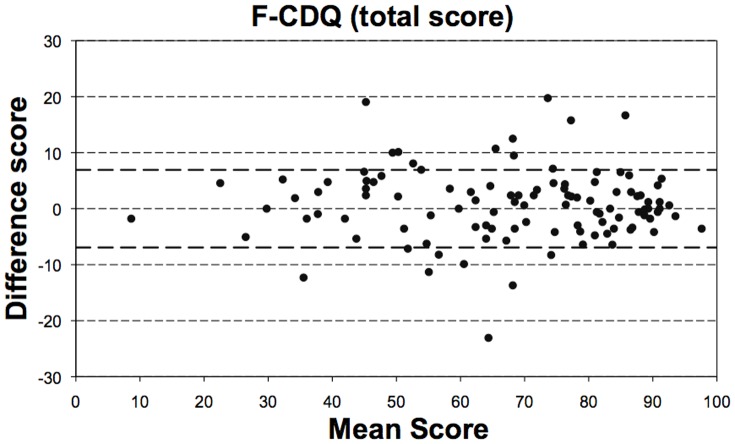
Bland and Altman graphical analysis of the reproducibility of the total score of the French version of the “Celiac Disease Questionnaire” (F-CDQ). Mean total score of the two F-CDQ assessments (baseline and retest 7 days later for patients who stated that their health status was unchanged) plotted against the difference of these two measures. The horizontal dotted lines indicate the mean difference ± 1 SD.

Of the 211 patients who completed the initial assessment, 144 completed the 7-day assessment, and of these, 112 stated that their CD-related health status had not changed significantly. The intraclass correlation coefficients for the 4 subscales were 0.88 to 0.94 (Table 2), indicating excellent reproducibility. The graphical method of Bland and Altman also revealed an excellent reproducibility throughout the range the F-CDQ total score (Figure 1) and the four subscales (not shown).

#### Internal consistency

The internal consistency was assessed by computing Cronbach's alpha coefficients using the data for all the 211 patients who completed the F-CDQ at baseline: the values were all very large (from 0.79 to 0.94; Table 2).

#### Structure

The initial first-order confirmatory factor analysis including only the four first-order factors (“Emotions”, “Social”, “Worries”, and “Gastrointestinal”) did not provide a good fit (CFI: 0.518 and RMSEA: 0.18). However, a model which involved the four first-order factors and one second-order factor (“general HRQoL”) showed a good fit, with a CFI of 0.946 and a RMSEA of 0.06. The factor loadings of items were 0.58–0.83, except for “restricted sexual activity” which loaded 0.30 on the “Social” factor.

Rasch model analysis also provided evidence for the unidimensionality of the four subscales, and only a single item from the “Social” subscale did not fit the model (Table 3). The continuum of the latent trait was better covered in the “Emotion” subscale (–0.87 to +0.67) than in the three other subscales. There were very few redundant items with very similar difficulties in any of the four subscales. This analysis also demonstrated that there was no significantly differential item functioning for age or gender.

**Table 3:** Rasch analysis of the four subscales of the French version of the “Celiac Disease Questionnaire” (F-CDQ).

Subscale	Short description of F-CDQ item	Location	Fit of item	
		(difficulty) *	Chi2	P
Emotion				
F-CDQ-16	“tearful or upset”	−0.87	5.65	0.06
F-CDQ-21	“satisfied, happy or pleased with personal life”	−0.30	2.2	0.3
F-CDQ-10	“depressed or discouraged”	−0.22	5.1	0.08
F-CDQ-14	“relaxed and free of stress”	0.04	0.57	0.75
F-CDQ-3	“frustrated, impatient or restless”	0.21	0.18	0.92
F-CDQ-6	“energy”	0.48	1.91	0.39
F-CDQ-2	“physically tired or exhausted”	0.67	2.98	0.23
Gastrointestinal				
F-CDQ-19	“nausea or retching”	−0.34	1.45	0.48
F-CDQ-17	“burping and belching”	−0.24	1.21	0.55
F-CDQ-5	“loose bowel movements”	−0.10	2.78	0.25
F-CDQ-1	“sudden urge for a bowel movement”	−0.03	2.35	0.31
F-CDQ-8	“stomach pains or cramps”	−0.01	5.37	0.07
F-CDQ-13	“feeling of incomplete bowel evacuation”	0.17	5.36	0.07
F-CDQ-11	“bloating or flatulence”	0.55	2.47	0.29
Worries				
F-CDQ-12	“afraid of getting cancer”	−0.19	1.58	0.45
F-CDQ-28	“fear of medical examination”	−0.15	1.09	0.58
F-CDQ-7	“passed celiac disease to children”	−0.14	1.04	0.59
F-CDQ-27	“diagnostic delay”	−0.10	2.66	0.24
F-CDQ-26	“lack of expertise from doctors”	−0.097	3.70	0.16
F-CDQ-25	“problems with social security or health insurance”	0.27	2.74	0.25
F-CDQ-24	“burdened by the cost and time required for the diet”	0.41	3.62	0.16
Social				
F-CDQ-18	“restricted sexual activity”	−0.23	4.48	0.11
F-CDQ-20	“lack of understanding from family or friends”	−0.16	1.03	0.60
F-CDQ-9	“recreational activities”	−0.098	1.39	0.50
F-CDQ-22	“lack of understanding from colleagues or managers”	−0.065	5.67	0.06
F-CDQ-4	“refused invitations for dinner”	−0.008	7.61	0.02 **
F-CDQ-23	“impeded studies or professional career”	0.18	4.89	0.08
F-CDQ-15	“isolated or excluded”	0.37	0.52	0.77

*: location of the items on the related subscale continuum as estimated by the Rasch model (items with negative values are more difficult to endorse than those with a positive value).

**: a single item did not adequately fit the Rasch model.

Item-subscale correlations were computed, and all coefficients were ≥0.40 (“Emotions”: 0.61–0.85; “Social”: 0.56–0.75; “Worries”: 0.44–0.61; and “Gastrointestinal”: 0.42–0.67).

We explored the relationships between the subscales of the F-CDQ and related conceptual dimensions of the MOS-SF36 and the HAD questionnaires (Table 4). All correlations coefficients were significant (P<0.0001). However, the magnitude and the direction of the associations were more evident for the related health domains of the compared health-related measures. Indeed, “Emotions” subscale of the F-CDQ was highly related to “Vitality”, “Role-Emotional” and “Mental health” subscales of the MOS-SF36, and both the “Depression” and “Anxiety” subscales of the HAD. Also, the strongest association of “Social” subscale of the F-CDQ was documented with “Social functioning” subscale of the MOS-SF36. As expected “Worries” subscale was mostly associated with “Mental health” and “Anxiety” subscales of the MOS-SF36 and the HAD, respectively. The strongest association of “Gastrointestinal” subscale was observed with “Bodily pain” of the MOS-SF36.

**Table 4:** Associations between the French version of the “Celiac Disease Questionnaire” (F-CDQ) and the MOS-36 and the HAD questionnaires.

F-CDQ subscales *					
	Emotions	Social	Worries	Gastrointestinal	Total score
MOS-SF36 subscales					
Physical function	0.46	0.38	0.33	0.42	0.47
Role-Physical	0.58	0.56	0.36	0.49	0.59
Bodily pain	0.58	0.53	0.48	0.63	0.65
Vitality	0.81	0.56	0.46	0.50	0.69
General health	0.61	0.51	0.50	0.55	0.64
Role-Emotional	0.69	0.49	0.37	0.45	0.59
Social functioning	0.75	0.68	0.54	0.49	0.73
Mental health	0.88	0.58	0.58	0.50	0.75
HAD					
Depression	−0.71	−0.54	−0.46	−0.45	−0.64
Anxiety	−0.74	−0.48	−0.60	−0.47	−0.68

MOS-SF36: medical outcome study short-form 36 items; HAD: hospital anxiety and depression scale; *: Spearman correlation coefficients. Negative correlations coefficients were expected between F-CDQ subscales scores and the HAD as these two instruments score in the opposite directions. P<0.0001 of all correlations coefficients.

The scores of all four subscales of the F-CDQ and total score correlated well with self-reported severity of CD and the number of associated CD manifestation (Table 5); this provides further evidence of convergent validity. Female sex was associated with significant worse score for the “Emotion” subscale. Patients with recently diagnosed CD (0–5 years) had significantly worse scores for three of the four subscales (except for “Emotions”) and total F-CDQ score.

**Table 5:** Association of the subscale scores for the French version of the “Celiac Disease Questionnaire” (F-CDQ) with gender, self-assessment of celiac disease severity and number of disease manifestations.

F-CDQ subscales	Gender			Disease severity score *			Number of celiac disease manifestations				
	Men	Women	P	<4	≥4	P	0 (N = 81)	1–2 (N = 27)	3–4 (N = 55)	≥5 (N = 48)	P
Emotions	61.6 (22.8)	51.5 (21.2)	0.006	59.8 (20)	43.5 (20.5)	0.0001	65.1 (20.4)	55.6 (17.8)	49.8 (18.7)	37.8 (18.9)	0.0001
Social	77.8 (24.1)	75.1 (23.5)	0.51	83.4 (18.1)	62.2 (25.8)	0.0001	86.2 (17)	79.8 (21.8)	74.2 (21.8)	57.4 (25.3)	0.0001
Worries	69 (25.5)	64.4 (22.1)	0.23	69.7 (21)	58.7 (23.3)	0.0008	74.3 (19)	67.7 (20.7)	62.6 (21.9)	52 (24.5)	0.0001
Gastrointestinal	72 (18.7)	68.2 (19.3)	0.24	76.1 (14.4)	55.8 (19.5)	0.0001	81.7 (12.1)	72.9 (16.7)	66.8 (14.8)	47.8 (15.4)	0.0001
Total score	70.1 (19.7)	64.8 (18)	0.09	72.3 (14.6)	55.1 (18.6)	0.0001	76.7 (13.7)	69.6 (15.5)	63.4 (15.1)	48.7 (16.9)	0.0001

*: self-reported disease severity assessed on a visual analogue scale (0–10) with 10 indicating the most severe disease; the F-CDQ scores were normalised, ranging from 0 (worst score) to 100 (best score) for each subscale and the total F-CDQ score.

#### Responsiveness

The responsiveness of the F-CDQ was studied at 6 months (Table 6). Consistent with the absence of intervention in this cohort, most of the patients (104 of 155) declared that their health status had remained in stable since the baseline assessment. An effect size from –0.03 to –0.54, according to the four subscales and total score, was observed for those patients who stated that their health status was worse at 6 months than baseline. Conversely, the effect sizes of the subscales and the total score of the F-CDQ questionnaire were from +0.01 to +0.37 for those who stated that their health had improved at 6 months. Overall, an effect size ≥0.20, generally considered to indicate a clinically important change, was observed in the “Emotions” and “Worries” subscales for patients who improved, and in “Emotions”, “Social”, “Gastrointestinal” subscale scores and the total score in patients who deteriorated.

**Table 6:** Sensitivity to change of the French version of the “Celiac Disease Questionnaire” (F-CDQ).

F-CDQ subscales	Improved after 6 months (N = 29)		Unchanged (N = 104)		Deteriorated after 6 months (N = 22)	
	Mean difference (SD)	Effect size	Mean difference (SD)	Effect size	Mean difference (SD)	Effect size
Emotions	7.1 (16.0)	0.36	0.1 (12.6)	0.01	–6.0 (14.5)	–0.25
Social	0.3 (13.2)	0.01	1.8 (11.3)	0.08	–5.7 (14.0)	–0.22
Worries	6.0 (16.4)	0.23	2.7 (12.9)	0.12	–0.7 (15.1)	–0.03
Gastrointestinal	0.3 (16.8)	0.02	–1.2 (12.3)	–0.06	–10.3 (14.4)	–0.54
Total score	3.4 (10.9)	0.18	0.9 (7.9)	0.05	–5.7 (11.0)	–0.27

## Discussion

The F-CDQ is rapid to complete and was well accepted by patients with CD. The proportion of missing items was less that 5%, which we considered to be acceptable, with the exception of one item. This item is related to the risk of transmitting CD to children and may not have been understood by some patients (some patients may not know that there is a genetic basis to the susceptibility to CD). However, a more plausible explanation is related to the fact that 93 subjects (44.1%) did not have children and that we omitted, as in the original instrument, to allow a “not relevant” option to this item; this should be definitely added to the questionnaire. There was no or minimal (<5%) floor or ceiling effects for three of the four subscales and the total score; for the “Social” subscale the ceiling effect was 15.7%. All patients who participated in our study had biopsy-proven CD, and this compares favourably with other studies many of which involved contacting patients through support groups and may therefore have been contaminated by “gluten-sensitive patients” [10]. Our study was limited to adult CD patients and therefore is uninformative as to whether the F-CDQ is suitable for children. The F-CDQ scores for our patient population were similar to those obtained in the study by Häuser et al. [14] and are entirely consistent with CD having a significant negative impact on HRQoL. Here also, we document that the HRQoL of CD patients was significantly worse than that of the French general population as assessed with age and gender standardized MOS-SF36 scores: the difference was between 0.3 to 0.8 SD for the eight subscales.

We found that the F-CDQ has good psychometric properties. Reliability was excellent. The four dimension structure of the original CDQ was confirmed by the factorial analysis. Also, Rasch analysis demonstrated the unidimensionality of each of the four subscales and documented the locations (difficulty) of items on the continuum of the measured HRQoL. Only a few items appeared to be possibly redundant. Both convergent and discriminant validity were similar to those of the original CDQ. Despite the absence of therapeutic intervention in these patients who were already on a GFD, the F-CDQ was able to detect significant clinical changes (ES ≥0.20) in those patients who stated that their health had changed (improvement or deterioration) after 6 months; the responsiveness of the CDQ may be greater shortly after GFD introduction for patients diagnosed recently.

As all of the translators who participated in the adaptation process of the F-CDQ were living in France this may limit the generalizability of its use in other francophone countries (i.e. Canada, Belgium, etc.).

The CDQ covers domains of particular relevance to patients with CD, including gastrointestinal symptoms, sexuality and financial issues. Indeed, this specific instrument was carefully developed with a special attention given to collecting the patients' perceptions of their disease. Another specific quality of life survey was recently developed by Dorn et al. [15] for patients with CD: this questionnaire (celiac disease quality of life survey: CD-QOL) comprises 20 statements that were similarly compiled from the views of patients with CD explores four subscales (“Limitations”, “Dysphoria”, “Health concerns”, and “Inadequate treatment”). It displays good reliability and construct validity; however, its responsiveness was not documented. An Italian version of this questionnaire has been recently published [16]. The conceptual framework may differ between the CDQ and the CD-QOL, the latter being based on a needs-based model that attempts to cover the attitudes and perceptions of individuals with CD. However, the two instruments have not been formally compared.

## Materials and Methods

### Administered questionnaires

#### Celiac Disease Questionnaire (CDQ)

The CDQ is a specific HRQoL instrument that comprises 28 items and explores four health dimensions (each with 7 items): “Emotions” (i.e. “How often during the last two weeks have you felt frustrated, impatient or restless?”), “Social” (i.e. “How many times during the last two weeks did you feel isolated from or excluded by others due to your celiac disease?”), “Worries” (i.e. “How many times during the last two weeks did you feel burdened by the expenses and time required obtaining gluten-free food?”), and “Gastrointestinal” (i.e. “How often during the last two weeks have your bowel movements been loose?”). It is a self-administered questionnaire and takes about 5 minutes to complete. The answers are provided on ordinal 7-point Likert's scales evaluating frequency or severity according to the item. The time frame addressed by the questionnaire is the previous 2 weeks. Missing values were imputed by the personal mean score method (the mean of the answers to a given dimension if the number of missing values did not exceed three for this dimension), although this was not planned by Häuser et al. in their original paper [14], [17].

The scoring system is simple and the instrument provides a score for each dimension as the sum of the corresponding items ranging from 7 (the worst score) to 49 (the best score) for each subscale, and a global HRQoL score as the sum of the four subscales (28–196; 49×4). To facilitate interpretation, the four dimensions and the global HRQoL scores reported herein are normalized to a 0 (the worst score) – 100 (the best score) range.

#### Medical Outcome Study Short-Form 36 (MOS-SF36)

The MOS-SF36 comprises 36 items assessing eight dimensions (physical functioning, physical role, bodily pain, general health, vitality, social functioning, emotional role, and mental health), each on a scale from 0 (worst score) to 100 (best score), with higher values representing better health status [5]. The scores were studied both as raw scores and as age- and sex-adjusted standardised scores using the French general population reference values for age (10-year interval groups) and gender, and expressed as standard deviations [18].

#### Hospital Anxiety and Depression scale (HAD)

The HAD is a short (14 items) self-assessment scale for detecting states of anxiety and depression that scores from 0 (best score) to 21 (worst score) [19]. We tested the hypothesis that patients with more symptoms, and patients who declared having more severe disease, would have higher subscale and total F-CDQ scores (worse HRQoL).

#### Visual analogue scale (VAS)

Celiac disease severity was self-assessed on a VAS that could range from 0 to 10, with 10 indicating the most severe disease.

### Methods and statistical analysis

#### Development of the French version of the CDQ

Cross-cultural adaptation of the CDQ was obtained by following the international guidelines to keep semantic, experiential and conceptual equivalence in translation [20], [21].

Three independent translations from German to French were produced allowing detection of errors and divergent interpretations of items with ambiguous meaning in the original instrument. The translators were aware of the purpose of the process and the concepts involved in the instrument, to favour idiomatic and conceptual rather than literal equivalence between the two versions of the questionnaire, and to render the intended measurement more reliable. Two out of the three translators were French native speakers and all were living in France (none were from francophone countries such as Canada or Belgium). Two back translations were produced by two independent translators whose mother tongue was German to amplify discrepancies in the first translation and to verify accuracy. After committee review of translations and back translations, a preliminary French version of the CDQ (F-CDQ) was obtained: the committee was constituted of the translators, gastroenterologists, methodologists experienced in cross cultural adaptation of quality of life questionnaires, and patients.

In a pre-test step the questionnaire was completed by ten patients with CD; the patients were then interviewed to explore the acceptability, comprehension and readability of the questionnaire (face validity); this resulted in the reformulation of a very small number of items, and the reformulations were validated by the committee (Appendix S1). In a final step the questionnaire was submitted to Dr Häuser (the author of the original instrument) who did not suggest any further modification.

#### Validation process

Standard descriptive statistics (mean, SD and range) were computed to display the score distributions for both separate items and dimensions of the instruments. Floor and ceiling effects, the percentage of non response, and item-scale correlations were examined.

Reliability was assessed by a test-retest method at 7 days. A transition question was administered at the time of the second administration and patients who declared that their health status changed (improved or deteriorated) between the two assessments were excluded from the reproducibility analysis. Intraclass correlation coefficients (ICC) were computed for the four subscales and the total score. An ICC of more than 0.80 indicated excellent reproducibility, one between 0.61 and 0.80 moderate reproducibility, and one between 0.41 and 0.60 fair reproducibility [22]. Internal consistency of the four subscales and the total score was assessed with Cronbach's alpha coefficients [23]; a value of 0.70 or higher is considered to indicate adequate internal consistency. A graphical analysis, by the Bland and Altman method, was also performed [24].

We used confirmatory factor analysis (CFA) to examine the factor structure of the F-CDQ. The aim of CFA, unlike that of exploratory factor analysis, is to test a hypothesized factor structure (here that of the original four dimensional CDQ) and to assess its fit to the data. The comparative fit index (CFI), and the root mean square error of approximation (RMSEA), were used to assess the fit of the model. A CFI of 0.90 or higher and a RMSEA of 0.08 or lower are usually considered to show a good fit [25]. Construct validity was also investigated by computing Spearman's correlation coefficients for convergent and divergent validity contrasting the results obtained with the F-CDQ and the two other self-administered instruments completed at baseline (the MOS-SF36 and the HAD).

In addition to the evaluation of the psychometric properties of the F-CDQ according to classical test theory, we also performed additional analysis using Rasch model analysis [26]. According to this model, the value of a subject's characteristics on the measured trait (underlying latent trait: quality of life) is a function of both the responses to the items and the difficulty of the items (estimated by the position of the item on an interval-level scale). We examined the fit of the data to the predictions of the model.

We assessed the responsiveness of the F-CDQ by retesting after 6 months. The patients were asked to complete the F-CDQ again, and answer a transition question (with three possible answers: the same or almost the same, better, or worse) about whether or not there had been any change in health status related to CD between entry into the study and the 6-month assessment. Effect size coefficients were computed for the four subscale scores and the total score of the F-CDQ, both for patients who noted an improvement or a deterioration in their health status. Thresholds of 0.2, 0.5 and 0.8 correspond to small, moderate and large changes, respectively [27].

Statistical analyses were performed using the Statistical Analysis System version 9 for Windows (SAS institute, Cary, NC). RUMM2020 software (Rumm Laboratory, Perth, Western Australia) was used for Rasch analysis.

### Patients and study design

This study was conducted in a single tertiary care referral centre. All adult (>18 years old) patients followed by the referral centre for celiac disease were invited to participate. All patients included in the study had biopsy-proven CD (duodenal biopsy showing evidence of villous atrophy and intraepithelial lymphocyte infiltrate) and specific antibodies.

The study consisted of a surface-mailing survey. A booklet comprising questions about socio-demographics characteristics and history of CD, the F-CDQ, and the HAD and the MOS-SF36 questionnaires were sent to each patient's home. A second questionnaire was sent to all patients 7 days later and a third F-CDQ questionnaire was sent 6 months later to those patients who had returned at least one of the first two questionnaires.

### Ethics statement

The final protocol was reviewed and approved by our institutional review board and an independent research ethics committee (Conseil d'Ethique Necker – Enfants Malades (CENEM) and CPP Ile de France 2). The return of the questionnaire booklet was considered to represent an implicit consent to participate in the study. The purpose of the study was explained in an accompanying letter, also included with the re-test and the follow-up measure at 6 months.

## Conclusions

There is growing interest in assessing the outcome of CD in a more adapted way, given the chronic nature of the disease. Patient reported outcomes have gained a wide acceptance and are highly valued by patients. The development of a self-administered HRQoL instrument that specifically captures the perceptions and concerns of individuals with CD is an important step forward in care of these patients. An Italian version of the CDQ has recently been published [28] and the development of other cross-culturally validated versions of this questionnaire will be useful both for cross-national surveys and for evaluating the benefits of any new therapeutic interventions.

## Supporting Information

Appendix S1 **Questionnaire Maladie Cœliaque.**
(DOC)
