# Supplementary material for: Validation of a French Version of the Quality of Life “Celiac Disease Questionnaire”
Source: PLoS One. 2014 May 2;9(5):e96346. doi: 10.1371/journal.pone.0096346 (PMC4008531; doi:10.1371/journal.pone.0096346)
Supplement: Appendix S1 — Questionnaire Maladie Cœliaque. (DOC) [file pone.0096346.s001.doc]

**Questionnaire Maladie Cœliaque**

Nous souhaiterions savoir comment vous vous êtes senti(e) au cours des 2 dernières semaines. Ce questionnaire porte sur les symptômes de la maladie cœliaque, sur la façon dont vous vous êtes senti(e) en général, et sur votre moral.

Veuillez lire attentivement chaque question et cocher la réponse (une seule) décrivant le mieux votre état au cours des 2 dernières semaines.

*Adaptation française du « Celiac Disease Questionnaire » (CDQ). Häuser et al. 2007. V1.0. Pouchot et al., 2013*

**********************

1. Au cours des 2 dernières semaines, avez-vous été gêné(e) par un besoin pressant d’aller à la selle ?
2. Au cours des 2 dernières semaines, de manière générale, vous êtes-vous senti(e) physiquement fatigué(e) ou épuisé(e) ?
3. Au cours des 2 dernières semaines, de manière générale, vous êtes vous senti(e) irritable ou énervé(e) ?
4. Au cours des 2 dernières semaines, avez-vous évité ou refusé une invitation à manger chez des amis ou de la famille à cause de votre maladie cœliaque ?
5. Au cours des 2 dernières semaines, vos selles ont-elles été très molles voire liquides ?
6. Au cours des 2 dernières semaines, de manière générale, avez-vous eu :
7. Au cours des 2 dernières semaines, avez-vous été inquiet(ète) à l’idée d’avoir transmis ou de pouvoir transmettre la maladie cœliaque à vos enfants ?
8. Au cours des 2 dernières semaines, avez vous été gêné(e) par des douleurs (crampes) au ventre ?
9. Au cours des 2 dernières semaines, avez-vous eu des difficultés dans vos loisirs ou vos activités sportives à cause de la maladie cœliaque ?
10. Au cours des 2 dernières semaines, de manière générale, vous êtes-vous senti(e) déprimé(e) ou découragé(e) ?
11. Au cours des 2 dernières semaines, avez-vous été gêné(e) par des ballonnements du ventre ou par l’émission de gaz (péter) ?
12. Les personnes atteintes d’une maladie cœliaque ont parfois des inquiétudes et des angoisses liées à leur maladie. Au cours des 2 dernières semaines, vous êtes-vous senti(e) inquiet(ète) ou angoissé(e) à l’idée de développer un cancer à cause de la maladie cœliaque ?
13. Au cours des 2 dernières semaines, avez-vous été gêné(e) par l’impression de ne pas avoir entièrement évacué vos selles ?
14. Au cours des 2 dernières semaines, de manière générale, vous êtes-vous senti(e) calme et détendu(e) ?
15. Au cours des 2 dernières semaines, avez vous eu le sentiment d’être différent(e) des autres ou bien d’être exclu(e) à cause de la maladie cœliaque ?
16. Au cours des 2 dernières semaines, de manière générale, vous êtes vous senti(e) déstabilisé(e) ou au bord des larmes ?
17. Au cours des 2 dernières semaines, avez vous été gêné(e) par des remontées ou renvois (roter) ?
18. Au cours des 2 dernières semaines, votre maladie cœliaque a-t-elle gêné votre activité sexuelle ?
19. Au cours des 2 dernières semaines, avez-vous été gêné(e) par des nausées ou des envies de vomir ?
20. Au cours des 2 dernières semaines, avez-vous souffert d’un manque de compréhension de vos proches (famille ou amis) concernant votre maladie cœliaque ?
21. Au cours des 2 dernières semaines, de manière générale, vous êtes vous senti(e) ?
22. Au cours des 2 dernières semaines, avez-vous souffert d’un manque de compréhension de vos collègues de travail ou de vos supérieurs concernant votre maladie cœliaque ?
23. Au cours des 2 dernières semaines, vous êtes-vous senti(e) pénalisé(e) dans vos études ou dans votre carrière professionnelle par votre maladie cœliaque ?
24. Au cours des 2 dernières semaines, vous êtes vous senti(e) gêné(e) par les dépenses supplémentaires ou le temps pris pour le régime sans gluten ?
25. Au cours des 2 dernières semaines, avez-vous été gêné(e) par des problèmes de prise en charge ou de remboursement des frais pour les aliments sans gluten ou les autres traitements de la maladie cœliaque (sécurité sociale ou assurance) ?
26. Au cours des 2 dernières semaines, avez-vous souffert d’un manque de connaissance sur la maladie cœliaque des médecins qui vous prennent en charge ?
27. Au cours des 2 dernières semaines, avez-vous été inquiet(ète) à l’idée que votre maladie cœliaque ait été diagnostiquée trop tard ?
28. Au cours des 2 dernières semaines, avez vous été angoissé(e) par les examens nécessaires pour votre maladie cœliaque (prise de sang ou endoscopie digestive) ?

******************

*Modalités de réponse* :

- Q1-Q5, Q7, Q8, Q10-Q17, Q19, Q20, Q22-Q28 : (1) tout le temps, (2) la plupart du temps, (3) souvent, (4) de temps en temps, (5) rarement, (6) presque jamais, (7) jamais
- Q6 : (1) pas du tout d’énergie, (2) très peu d’énergie, (3) peu d’énergie, (4) de l’énergie, (5) pas mal d’énergie, (6) beaucoup d’énergie, (7) plein d’énergie
- Q9 : (1) très grandes difficultés (ou activités impossibles), (2) grandes difficultés, (3) assez de difficultés, (4) quelques difficultés, (5) peu de difficultés, (6) presque pas de difficultés, (7) aucune difficulté, la maladie cœliaque n'a pas restreint mes loisirs ou mes activités sportives
- Q18 : (1) aucune activité sexuelle en raison de la maladie cœliaque, (2) très forte gêne, (3) forte gêne, (4) gêne, (5) peu de gêne, (6) presque pas de gêne, (7) aucune gêne, *non concerné*
- Q21 : (1) la plupart du temps très insatisfait(e), très malheureux(se), (2) généralement insatisfait(e) ou malheureux(se), (3) assez souvent insatisfait(e) ou malheureux(se), (4) ni satisfait(e) ou insatisfait(e), ni heureux(se) ou malheureux(se), (5) assez souvent satisfait(e) ou heureux(se), (6) généralement satisfait(e) ou heureux(se), (7) la plupart du temps très satisfait(e), très heureux(se)
